# Supplementary material for: Polyketide synthase gene clusters in the ericoid mycorrhizal fungus Oidiodendron maius: genome-wide prediction, expression profiling, and functional characterization of knockout mutants
Source: Front Plant Sci. 2026 Sep 16;17:1924430. doi: 10.3389/fpls.2026.1924430 (PMC13623903; doi:10.3389/fpls.2026.1924430)
Supplement: Supplementary file 1 [file SupplementaryFile1.zip › Supplementary Materials/Supplementary Folder 1.zip!/Oidma1_AssemblyScaffolds_Repeatmasked/knownclusterblast/cluster22/jgi.p_Oidma1_126176_mibig_hits.html]

| MIBiG Protein | Description | MIBiG Cluster | MiBiG Product | % ID | % Coverage | BLAST Score | E-value |
| --- | --- | --- | --- | --- | --- | --- | --- |
| AGO65988.1 | putative\_cytochrome\_p450 | BGC0000992 | Fusaridione\_A\_biosynthetic\_gene\_cluster | 38 | 84.2105263158 | 272 | 1.3e-72 |
| BAD83681.1 | cytochrome\_P-450 | BGC0000012 | Alternapyrone\_biosynthetic\_gene\_cluster | 35 | 98.6354775828 | 269 | 6.6e-72 |
| ACD75398.1 | cytochrome\_P450\_monooxygenase\_CYP52-M1 | BGC0001274\_c5 | Non-acetylated\_open-chain\_sophorolipid\_/\_diacetylated\_lactonic | 35 | 85.3801169591 | 245 | 1e-64 |
| AFV52138.1 | P450\_monooxygenase | BGC0000081 | Kedarcidin\_biosynthetic\_gene\_cluster | 26 | 84.9902534113 | 127 | 5.3e-29 |
| ABP55138.1 | cytochrome\_P450 | BGC0000150 | Sporolide\_biosynthetic\_gene\_cluster | 24 | 84.9902534113 | 113 | 6.1e-25 |
| AAM78009.1 | cytochrome\_P-450 | BGC0000112 | Neocarzinostatin\_biosynthetic\_gene\_cluster | 26 | 73.2943469786 | 109 | 1.5e-23 |
| AAQ17113.2 | putative\_P450\_hydroxylase | BGC0001008 | Maduropeptin\_biosynthetic\_gene\_cluster | 26 | 81.4814814815 | 108 | 3.3e-23 |
| AAL06697.1 | P450\_hydroxylase | BGC0000965 | C-1027\_biosynthetic\_gene\_cluster | 24 | 86.5497076023 | 102 | 1.8e-21 |
| ALU98459.1 | cytochrome\_P450 | BGC0001397 | Lidamycin\_biosynthetic\_gene\_cluster | 24 | 86.5497076023 | 102 | 1.8e-21 |
| CAQ18837.1 | putative\_cytochrome\_P450 | BGC0000954 | Ajudazol\_biosynthetic\_gene\_cluster | 25 | 80.8966861598 | 94 | 5e-19 |
| BAD86796.1 | putative\_P450 | BGC0000286 | Viguiepinol\_biosynthetic\_gene\_cluster | 26 | 72.3196881092 | 77 | 3.7e-14 |
| AAC49196.1 | putative\_p450\_monooxygenase | BGC0000152 | Sterigmatocystin\_biosynthetic\_gene\_cluster | 25 | 65.4970760234 | 62 | 2.1e-09 |
